# Spectral correlations in a random distributed feedback fibre laser

**DOI:** 10.1038/ncomms15514

**Published:** 2017-05-18

**Authors:** Srikanth Sugavanam, Mariia Sorokina, Dmitry V. Churkin

**Affiliations:** 1 Aston Institute of Photonic Technologies, Aston University, Aston Triangle, Birmingham B4 7ET, UK; 2 Novosibirsk State University, Pirogova str. 2, Novosibirsk 630090, Russia

## Abstract

Random distributed feedback fibre lasers belong to the class of random lasers, where the feedback is provided by amplified Rayleigh scattering on sub-micron refractive index inhomogenities randomly distributed over the fibre length. Despite the elastic nature of Rayleigh scattering, the feedback mechanism has been insofar deemed incoherent, which corresponds to the commonly observed smooth generation spectra. Here, using a real-time spectral measurement technique based on a scanning Fabry-Pérot interferometer, we observe long-living narrowband components in the random fibre laser's spectrum. Statistical analysis of the ∼10^4^ single-scan spectra reveals a preferential interspacing for the components and their anticorrelation in intensities. Furthermore, using mutual information analysis, we confirm the existence of nonlinear correlations between different parts of the random fibre laser spectra. The existence of such narrowband spectral components, together with their observed correlations, establishes a long-missing parallel between the fields of random fibre lasers and conventional random lasers.

## Abstract (web summary)

The feedback mechanism in random fibre lasers has been insofar deemed incoherent. To reveal the dynamic evolution of the random fibre laser spectra, Sugavanam *et al*. use a real-time spectral measurement technique and observe long-lived narrowband components in the random fibre laser's spectrum.

Random lasers represent a new modality of lasing, wherein the conventional cavity configuration is replaced by a random arrangement of scatterers embedded in gain media. Multiple scattering events increase the mean free path of the photons within the gain media, leading to an avalanche generation of stimulated photons^1^ (the so-called ‘photonic bomb' effect). This principle has been very successfully employed in realizing lasing in different configurations of both strongly scattering^2^,^3^,^4^ and weakly scattering systems^5^,^6^. Random lasers offer a simple and natural platform for the study of photon propagation in disordered media, which in turn has led to the realization of practical solutions, such as speckle-free imaging^7^ and even highly miniaturized compact spectrometers^8^. A substantial body of research has come up recently, primarily motivated by the prospect of realization of high-brightness sources with significantly reduced design complexity (see, for example, refs 9, 10 for excellent reviews).

Another type of random laser that has been demonstrated recently is the random distributed feedback fibre laser^11^. Here, an optical fibre is used as the light transport medium, offering a natural mechanism for confinement and directionality of the lasing light. Lasing feedback is derived from weak Rayleigh scattering events occurring along the length of the fibre, amplified using distributed gain. The ready availability of telecommunications-grade active and passive elements, further motivated by the inherent benefits offered by an all-fibre platform and simplicity of design, has led to an explosion of research in this area, resulting in the realization of high-power random lasers over a broad wavelength range, with demonstrated efficiencies approaching quantum limits, and also in the envisioning of several practical applications including sensing and long-haul telecommunications (see recent review^12^ and references therein).

While significant progress has been made in the understanding of the underlying physics of random fibre lasers, there still remains a conundrum. It is known that in conventional random lasers based on strongly scattering systems, multiple scattering events can result in generation of closed loops within the medium that can sustain a coherent feedback^9^. These can result in a generation spectrum consisting of well-defined spectral peaks with high *Q*-factors, which follow the Schawlow-Townes linewidth relation^13^. This is in contrast with incoherent feedback random lasers, where in the absence of closed photon loops the generation spectrum is devoid of such narrow features and is smooth^14^. However, in some cases such spectral peaks may also appear in weakly scattering, diffusive systems^15^, and can be attributed to the intrinsic low-*Q* cavity resonances and the interaction between them^16^, or from amplified Levy flights of photons^6^,^17^.

The optical spectra of random fibre lasers have been conventionally reported to be of a smooth nature, a feature that has been commonly attributed to an underlying incoherent feedback mechanism. This is also in good agreement with the existing theoretical descriptions of the random fibre laser based on a wave kinetic approach under the consideration of fully incoherent nature of feedback^18^. A numerical model based on coupled nonlinear Schrödinger equations with an assumption of incoherent feedback^19^ reproduces the experimentally observed smooth spectrum as well.

However, Rayleigh scattering is elastic, i.e., scattering events are random in space but fully deterministic in both phase and amplitude^20^,^21^. Thus in principle, the intrinsic nature of Rayleigh scattering could result in a coherent type of the feedback, leading to the formation of extremely low-*Q* cavities that can give rise to narrow peaks in the generation spectrum. The existence of narrow spectral peaks have not been reported in random fibre lasers thus far and, consequently, it remains unclear if the coherent properties of the Rayleigh scattering affect the performances of random fibre lasers.

In this paper, we report the experimental observation of narrowband spectral components in a random fibre laser. We use a real-time spectral measurement technique based on a high resolution, fast scanning Fabry-Pérot interferometer (FPI) to reveal the dynamic evolution of the random fibre laser spectra. Near the generation threshold, the spectra are seen to comprise several narrowband components that are an order of magnitude narrower than the total spectral width of the generation. The real-time measurement methodology reveals the complex non-stationary nature of the generation spectrum and the millisecond order lifetimes of the narrowband components, and also allows for an in-depth statistical analysis of the spectral characteristics. While the Pearson correlation coefficient indicates the existence of correlations and anti-correlations between different parts of the spectra, an analysis of the mutual information content between the different spectral components reveals direct experimental evidence for the existence of a mutual dependence between them.

## Results

### Principle

**Figure 1:**
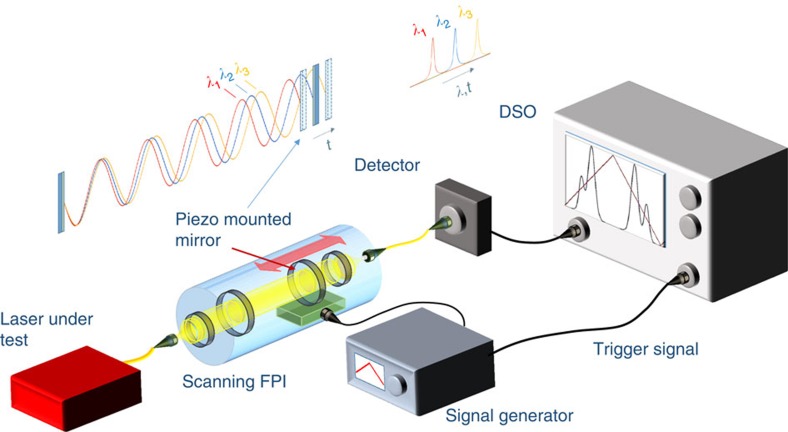
Real-time spectrum to time mapping using a scanning Fabry-Pérot interferometer (FPI). The transmission wavelength of an FPI is a function of its mirror interspacing. Hence, a fast scanning action aided by a piezo-mounted mirror results in a sampling of different wavelengths at different time instants—a spectrum-to-time mapping. The spectra can then be monitored in a continuous fashion using a digital storage oscilloscope (DSO), wherefrom the real-time spectral evolution can be reconstructed.

The scanning FPI-based real-time spectral measurement configuration for the schematically shown in Fig. 1. The spectrally selective transmission peak of the FPI can be tuned by scanning one of the mirrors in a periodic fashion, allowing for a spectrum-to-time mapping of the input radiation. The time to frequency mapping factor can be obtained using the relation between the free spectral range Δ*f*_FSR_ of the interferometer and the time Δ*t*_FSR_ taken to sweep through it as *δf*=(Δ*f*_FSR_/Δ*t*_FSR_)*δt*, where *δt* is the sampling rate of the oscilloscope (see Supplementary Note 1). The primary advantage offered by such FPIs is their high resolution, which has been successfully exploited for the spectral characterization of narrow bandwidth sources^22^. However, the fast scanning capabilities of such devices for the characterization of time-varying spectra have not been exploited so far. Piezo-enabled FPIs are capable of operating at frequencies ∼10^2^ Hz, corresponding to a spectral acquisition rate that is at least two orders of magnitude higher than conventional Czerny–Turner-based optical spectrum analyser (OSA) configurations. Hence, a scanning FPI can be used to recover non-averaged, dynamic spectra evolving over time scales comparable to the FPI scan rate. To facilitate real-time measurements, the output from the FPI is recorded in a continuous fashion using a digital storage oscilloscope, helping in the acquisition of a large number of spectra. These can now be used to recover the real-time spectral evolution, and also enable statistical studies of the underlying spectral dynamics. Here, the resolution and the scan rate of the FPI are 2 pm and 90 Hz respectively.

### Real-time spectral dynamics

**Figure 2:**
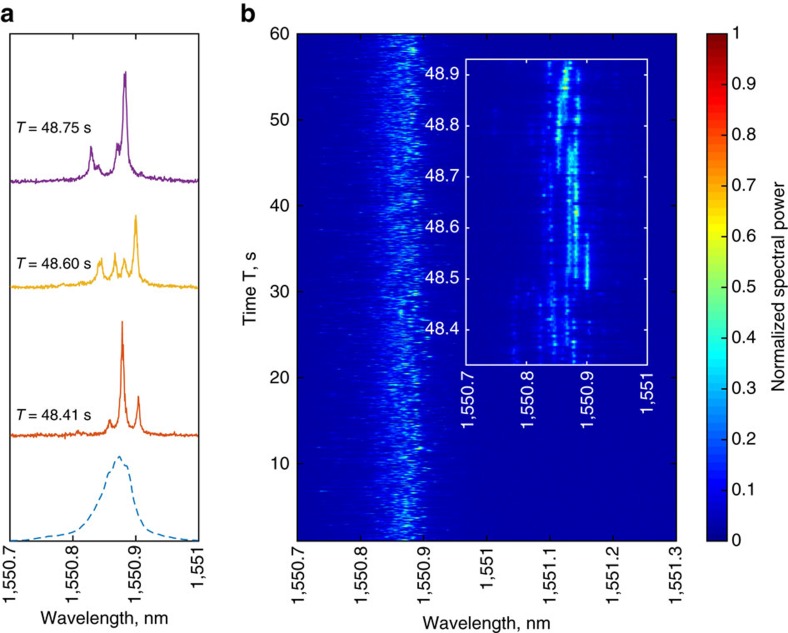
Real-time spectro-temporal evolution in the random fibre laser. (**a**) Single-scan spectra of the random fibre laser, seen to comprise several narrowband features. Blue dotted curve—average spectra. (**b**) Long term, real-time spectral evolution obtained from consecutive scans. Inset—evolution dynamics of the narrowband features shown in **a** that clearly reveals their millisecond order lifetimes.

The random fibre laser used in experiment is of a uni-directional, grating based, narrowband configuration, using Stimulated Raman Scattering (SRS) for gain and employing 40 km of standard telecommunications grade fibre for generating the feedback (see Methods and Supplementary Fig. 1). Threshold is realized near the pump power of 1.15 W, with typical output powers of the order of 10 mW near 1.20 W. The grating used is centred around 1550.8 nm, having a Super Gaussian profile with a spectral bandwidth of 0.2 nm. Just above the lasing threshold, the spectra of random fibre lasers are documented to be very noisy (e.g., see ref. 11), and this has been primarily attributed to the role of Stimulated Brillouin Scattering (SBS). However, conventional OSA measurements have insofar yielded almost no information about the underlying dynamics in this region of operation of the laser. Here, by using the FPI-based measurement method, the spectral dynamics of the random fibre laser just above threshold are revealed for the first time. Figure 2a shows several instances of the spectra acquired by the scanning FPI. These show that the random fibre laser spectra comprises several narrow-band spectral components in the generation, whose spectral widths are an order of magnitude narrower than the total generation width. The spectral widths of these components are beyond the resolution of the measuring configuration. The number of peaks are seen to vary over time along with their interspacing. The typically observed noisy OSA spectrum can thus also be attributed to the occurrence of this previously undiscovered underlying spectral dynamic, apart from SBS-related effects.

As the spectra are acquired in a continuous fashion, the evolution of the spectra over time can be obtained in a straightforward manner by stacking successive sweeps of the FPI one atop another (Fig. 2b and Supplementary Fig. 4). Here, spectral evolution in the random fibre laser is monitored in a continuous fashion over a time scale of 60 s, resulting in the acquisition of ∼10^4^ individual spectra at an effective sampling rate of 180 Hz—almost two orders of magnitude higher than conventional OSAs. Note that both forward and backward scanned FPI spectra are used in arriving at the spectro-temporal representation (see Methods and Supplementary Fig. 2). The two-dimensional spectro-temporal evolution reveals that the generated narrowband components survive over time scales of several hundred milliseconds, which is much longer than the typical time of flight over the 40 km fibre span. Further, as the spectra evolve at time scales larger than the acquisition rate of the FPI, the spectral measurements can be regarded to be of an instantaneous nature. The existence of narrowband components in conventional random lasers have been confirmed using streak camera-equipped spectrometers^23^, which were also adapted to spatio-temporally map time-dependent spectral dynamics^24^. However, such bulk spectrometric configurations are not suited for the case of the random fibre laser, which gives rise to quasi-continuous wave generation with temporally varying spectral dynamics. In this regard, the scanning FPI offers a straighforward way for the real-time spectral characterization of the random fibre laser. It clearly reveals the existence of narrow-band spectral components in the random fibre laser. The observed spectra are very similar (narrow peaks on the broad smooth background) to those observed and numerically simulated in diffusive random laser systems with coherent feedback^13^.

### Peak distribution statistics

**Figure 3:**
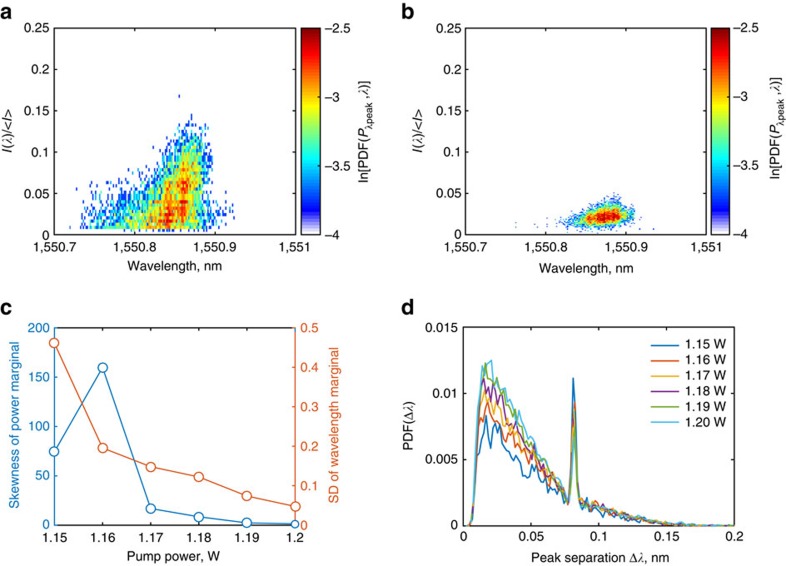
Peak distribution statistics. (**a**,**b**) Two-dimensional joint probability distributions of the peak wavelengths with respect to intensity (in logarithmic scale). (**c**) Skew and standard deviation (s.d.) of the power and wavelength marginals, respectively, showing how fluctuations in intensity and peak location decrease with increasing pump power. (**d**) PDF of the peak separations, showing a preferential spacing of 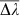
≈0.02 nm. The pronounced narrow peak arises due to SBS.

The spectro-temporal evolution of the random fibre laser (Fig. 2) show that several narrowband components occur in the generation spectrum, differing in their peak powers and spectral locations. The availability of a large spectral ensemble (∼10^3^ spectra) facilitates a wavelength-resolved statistical investigation of the occurrences of such peaks. Figure 3a,b show the two-dimensional, joint probability distribution functions 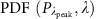, where *λ*_peak_ are the wavelength locations of the peaks, and 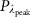 their corresponding powers, normalized by the mean power level of the output 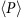. The PDFs highlight the marked difference in generation dynamics—the joint PDF obtained for the spectra acquired just above threshold (Fig. 3a) indicate spectral components with higher peak powers are more likely to occur towards the red end of the spectrum, eventually leading to a clustering of the peaks towards the red end of the spectrum at higher pump powers.

The stability of the generation can then be quantified in terms of the skewness of the power marginal, 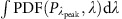 (Fig. 3c), which here is seen to decrease steadily towards zero with increasing pump power (also see Supplementary Fig. 5). The clustering of the spectral peaks in turn can be given by the standard deviation of the wavelength marginal 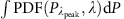. Note that the slightly lower value of the skewness at 1.15 W is due to intermittent generation dynamics that leads to a high value of zero-events in the marginal PDF.

Further insight can be obtained from the PDF of the peak interspacing, shown in Fig. 3d for various pump powers. The pronounced peak near the peak separation value of 0.08 nm is observed for low pump powers can be attributed to the SBS mechanism. This implies the generation of SBS-Stokes components, thus explaining the observed high-power red-shifted components in the joint PDF of Fig. 3a. The mode of the distribution 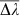 of Fig. 3d corresponding to the most probable peak spacing has a value of ≈20 pm, and is seen to be independent of the pump power. Indeed, several instances of the measured spectra (see Fig. 2b) show a well-defined peak separation approaching this value. The existence of a preferred interspacing is indicative of some form of underlying correlation between the modes.

### Spectral correlations in the random fibre laser

**Figure 4:**
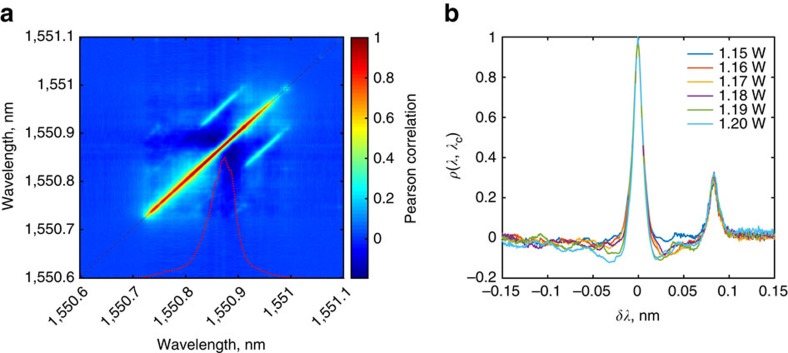
Spectral correlations in the random fibre laser. (**a**) The Pearson correlation coefficient matrix for *P*_p_=1.20W, and (**b**) its cross-section for different pump power values. Anti-correlations are observed at wavelength spacing 0.02 nm corresponding to 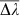.

The existence of correlations in the random fibre laser can be verified by calculating the Pearson's correlation coefficient, 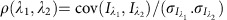. Similar approaches have been used in conjunction with the real-time spectral measurement based on Dispersive Fourier Transformation (DFT)^25^, to reveal underlying spectral correlations in supercontinuum generation^26^, and also correlations between the sidebands in conventional modulation instability^27^. As DFT-based spectral measurement can be used only for pulsed generation, the FPI-based configuration offers a complementary route for studying such correlations over a wide variety of continuous and quasi-continuous wave systems. Figure 4a shows the matrix of *ρ* values for *P*_p_=1.20 W, where the red overlay corresponds to the average spectrum obtained over the measured ensemble (also see Supplementary Fig. 6). Figure 4b gives the cross-sections *ρ*(*λ*, *λ*_*c*_) of this coefficient matrix, where *λ*_*c*_ is peak wavelength location corresponding to the averaged spectrum, and *δλ*≡*λ*−*λ*_*c*_. The secondary peak centred at 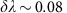 nm are indicative of the existence of SBS processes. But more importantly, the Pearson coefficient reveals an anti-correlation of dynamics for the wavelength spacing of ≈20 pm corresponding to the most probable spacing 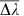 as shown in Fig. 3d. The observed anti-correlation can potentially be attributed to homogeneous gain dynamics. While Raman gain can be attributed to the contribution of multiple vibrational modes^28^ and can be regarded to be inhomogeneous, the gain bandwidths arising from the individual vibrational modes are much broader than 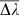. Thus, the observed spectral components in the random fibre laser observed compete for gain arising from a single vibrational mode, giving rise to the observed anti-correlated dynamics. Note that similar anti-correlated dynamics have also been observed in conventional random lasing configurations^29^, where the preference for a particular wavelength spacing in turn could arise from an interplay between gain competition and the spatial distribution of the narrowband components along random media. The estimation of the Pearson's correlation coefficient aided by the real-time FPI-based spectral measurement thus offers a simple, complementary and straightforward way of checking the existence of spectral correlations.

While the Pearson correlation coefficient helps to identify and quantify the extent of correlations, they are sensitive only to a linear relationship between the two variables under consideration. The existence of nonlinear dependencies between the generated spectral components can be revealed by the method of mutual information analysis. Mutual information was introduced by Claude Shannon in 1948 (ref. 30) as a fundamental concept of information theory, and is now widely used in telecommunications, networks, machine learning, as well as in physics of complex systems^31^,^32^,^33^,^34^. In particular, it has been used for analysis of time series laser dynamics^35^, where permutation entropy has been used for revealing the role of the different time scales. While permutation entropy (and statistical complexity) has been used to identify and compare characteristic timescales of the system, it cannot reveal the strength of interactions between the components. As statistical complexity qualifies the difference of the probability distribution of the system under consideration from one exhibiting a uniform distribution, thus characterizing the level of complexity of the system between order and complete randomness, while entropy serves as quantifier of information content—neither technique reveals the underlying processes and interaction (as in information exchange) in the system. Here we focus on complex interplay—nonlinear dependence between different spectral components. Thus, mutual information as a measure of intricate dynamics and information transfer between two variables is applied for analysis.

The mutual information for two variables *X* and *Y* is defined as


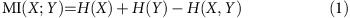


where 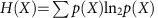 is the Shannon entropy of the corresponding variable. It has a meaning similar to Boltzmann entropy in physics and has been introduced as a measure of information or measure of uncertainty of the variable. Similarly, joint entropy *H*(*X*,*Y*) is defined for the joint ensemble of variables. To study the interference between two variables we use mutual information, defined as a measure of the amount of information that one random variable contains about another; in other words, it is the reduction in the uncertainty of one random variable due to the knowledge of the other. High mutual information indicates a large reduction in uncertainty; low mutual information indicates a small reduction; while mutual information between two random variables equals to zero only if the variables are independent.

**Figure 5:**
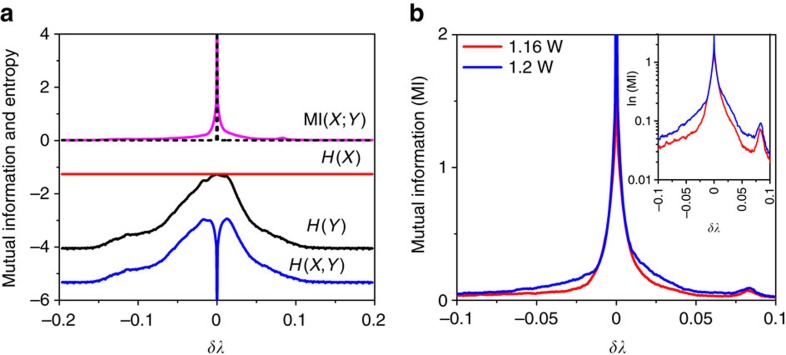
Mutual information analysis of random fibre laser spectra. (**a**) *H*(*X*) (red curve) and *H*(*Y*) (black) are the Shannon entropies calculated from each single-sweep spectra. Here *X* represents the intensity measured at wavelength *λ*_*c*_, the peak wavelength location of the time-averaged spectral dynamics. *H*(*X*,*Y*) (blue curve) is the corresponding joint entropy. The non-vanishing nature of mutual information (MI) (magenta), away from *δλ*≡*λ*−*λ*_*c*_=0 implies dependence between the components. For comparison, black dotted curve shows the mutual information for uncorrelated random variables. (**b**) MI plotted for two different pump powers (inset in log-scale). Compared to Fig. 4b, the non-zero MI indicates dependence between components, even if the Pearson correlation coefficient might be zero.

Figure 5a shows the mutual information between a spectral component at given wavelength *λ* and the peak wavelength location *λ*_*c*_ corresponding to the averaged spectrum (blue dotted curve in Fig. 2a), and its relation to the corresponding entropies of equation (1). One can see that mutual information has a strong peak 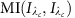 at the wavelength *λ*_*c*_, with its value decaying rapidly with the increasing distance *δλ*=*λ*−*λ*_*c*_. However, note that it does not go to zero, as is typically expected for two independent random variables (see black dashed line in Fig. 5a). In Fig. 5b we plot MI from two data sets for different pump powers (*P*_p_=1.16 W, *λ*_*c*_=1550.8 nm; *P*_p_=1.20 W, *λ*_*c*_=1550.9 nm). The SBS-contribution for *δλ*=0.08 nm is evident from these plots. Further, with the increase of peak power the slope of mutual information is smaller and we see wider region of non-zero mutual information, whereas the second peak of mutual information rises to higher values. This is also highlighted in logarithmic scale in the inset of Fig. 5b.

**Figure 6:**
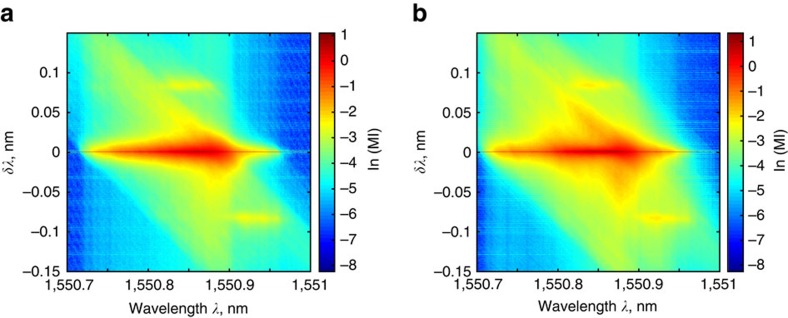
Mutual information contours. Value of the logarithm of MI over the spectra for (**a**) *P*_p_=1.16 W and (**b**) *P*_p_=1.20 W. Non-vanishing value of the MI across all spectral values indicates the existence of correlations over the whole-generation span.

Contour plots in Fig. 6a,b (plotted for *P*_p_=1.16 W and *P*_p_=1.20 W respectively) demonstrate the dynamic across different wavelengths. The non-vanishing nature of the mutual information indicates towards the existence of dependence between the different spectral components generated by the random fibre laser (also see Supplementary Fig. 7). Further, here we also see that an absence of correlation does not imply independence—although Pearson correlation coefficient returns a value of zero for certain wavelength separations, the mutual information has a finite non-zero value for the same separation, showing mutual dependence between the generated wavelength components. Indeed, the existence of correlations in the generation spectrum have been observed in experiment. The mutual information approach thus provides an indirect signature of the existence of correlations between different parts of the random fibre laser spectrum.

## Discussion

The real-time spectral measurement technique based on the FPI has allowed to observe individual narrowband spectral components in the radiation of the random fibre laser. This establishes an important parallel between random fibre lasers and conventional random lasers, where the occurrence of narrow-band components have been attributed to Levy flights, coherent feedback mechanisms and interactions between cold cavity resonances. The occurrence of such narrowband components also calls in for the re-examination of the underlying nature of generation in the random fibre laser. The time-dependent spectral evolution, together with the occurrences of anticorrelations, highlight the role of gain competition in the laser, which can potentially give rise to spatially dependent spectral dynamics^29^. Furthermore, MI analysis of the ∼10^4^ spectra has revealed a non-vanishing, nonlinear mutual dependence between different parts of the random fibre laser spectrum.

It is of interest to observe the possible manifestation of narrow-band spectral components in the time domain. Indeed, the assumption of fully incoherent feedback leads to the necessity of completely Gaussian statistics of amplitudes of frequency components and exponential statistics of total intensity dynamics^18^. However, the real temporal and statistical properties reveal that the intensity statistics deviates from exponential^36^. This could be an indication of influence of coherent nature of the feedback in the random fibre laser and, possibly, the manifestation of mutual correlations between narrow-band spectral components. The direct measurements of time dynamics of narrow-band spectral components is of interest in this regard and could be done via spectral pre-filtering of the radiation before making temporal measurements.

The findings above also serve to highlight the significant potential of the scanning FPI as a real-time spectral measurement tool. The order of magnitude improvement on both the scan rate and spectral resolution offered by the FPI over conventional OSAs allow for rapid acquisition of high-quality, high-resolution spectra. While they are limited in resolution and free-spectral range, they are compatible with general continuous and quasi-continuous wave sources, hence complementing existing well-established real-time spectral measurement methods like the DFT^25^. Furthermore, they allow a straightforward method to arrive at wavelength-resolved statistical estimates like Pearson correlations, and even other formulations of the two-point correlation functions^37^, which can serve to quantize the degree of coherence of the random fibre laser, and uncover possible occurrences of phase transitions as observed in conventional random lasers^38^,^39^.

The simplicity of configuration and ease of implementation of the random fibre laser has led to a proliferation of applications ranging from optical sensing to telecommunications. An understanding of the nature of such underlying correlations can have profound implications in the intelligent design of random fibre lasers geared towards applications, increasing their versatility even further.

## Methods

### Random fibre laser design

The random fibre laser used above is of a uni-directional, grating-based configuration. Forty kilometres of conventional Corning SMF-28e is used for the laser generation. A Super-Gaussian grating, centred at 1550.85 nm with 3 dB bandwidth of 0.2 nm and contrast of 12 dB, is used for filtered feedback. The laser is pumped using a 1,455 nm Raman laser. The outputs are connectorized with angle-connectors to avoid reflections. A double stage isolator is additionally spliced at the output to avoid spurious back-reflections (also see Supplementary Fig. 1).

### Spectral acquisition using the scanning FPI

A fibre pigtailed scanning FPI with a free spectral range of 340 GHz and Finesse 963 is used. The scanning of the FPI was controlled using a saw-tooth signal of frequency 90 Hz. The output of the FPI was monitored using a 17 MHz detector connected to a digital storage oscilloscope. The temporal sampling rate of 1.0 *μ*s, resulting in ∼3 points per 2 pm sufficiently resolved the individual spectral features in the generation. Both the FPI output and the sweep voltage signal were recorded simultaneously along two channels. As the FPI samples the spectra during both up and down sweeps, both sweep instances were used for the reconstruction of the spectro-temporal dynamics, resulting in an effective spectral acquisition rate of 180 Hz (see Supplementary Fig. 2). The spectra were registered with respect to the starting locations of the corresponding sweeps, and a simple flip-&-interleave routine was written to order the consecutive sweeps. The absolute location of the spectra was registered using a tunable laser source, and verified independently with conventional OSA measurements (see Supplementary Fig. 3 and Supplementary Note 1).

### Statistical analysis of peak distribution

The peak locations and their peak intensities were obtained from the FPI measured initial intensity optical spectra. The minimum peak separation parameter and the peak width parameter were set at 2 pm. The peak threshold level was set at 1/10th the height of the largest peak. The peak locations along their intensities were stored as an ordered pair, which were then used to obtain the two-dimensional histograms of Fig. 3 using MATLAB. The probability density function of the peak difference, Fig. 3d, was obtained using a simple matrix based routine as follows: let for instance, the peak locations obtained for a given spectral scan was (*p*_1_, *p*_2_, *p*_3_). Two matrices *A* and *B* were constructed such that


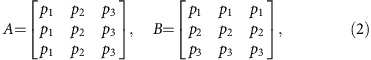


such that the absolute value of the upper (or lower) triangular matrix of the difference *C*=*A*−*B* yields all possible combinations of peak separations. The values of *C* from the different single-scan spectra were then collated to obtain the histograms of Fig. 3d as shown.

### Data availability

The data that support the findings of this study are available from the corresponding author upon reasonable request.

## Additional information

**How to cite this article:** Sugavanam, S. *et al*. Spectral correlations in a random distributed feedback fibre laser. *Nat. Commun.* **8,** 15514 doi: 10.1038/ncomms15514 (2017).

**Publisher's note**: Springer Nature remains neutral with regard to jurisdictional claims in published maps and institutional affiliations.

## Supplementary Material

Supplementary Information Supplementary Figures, Supplementary Note and Supplementary References
