# Supplementary material for: Spectral correlations in a random distributed feedback fibre laser
Source: Nat Commun. 2017 May 18;8:15514. doi: 10.1038/ncomms15514 (PMC5454388; doi:10.1038/ncomms15514)
Supplement: Supplementary Information — Supplementary Figures, Supplementary Note and Supplementary References [file ncomms15514-s1.pdf]

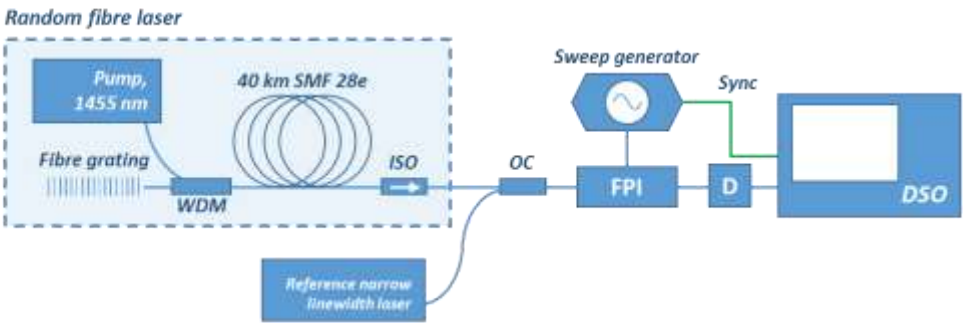

Supplementary Figure 1: Experimental configuration. WDM – wavelength division multiplexer, SMF – single mode fibre, OC – optical couper, FPI – scanning Fabry-Perot interferometer, D – photodetector, DSO – digital storage oscilloscope. The reference narrow linewidth laser helps in wavelength calibration and registration of the measured spectra.

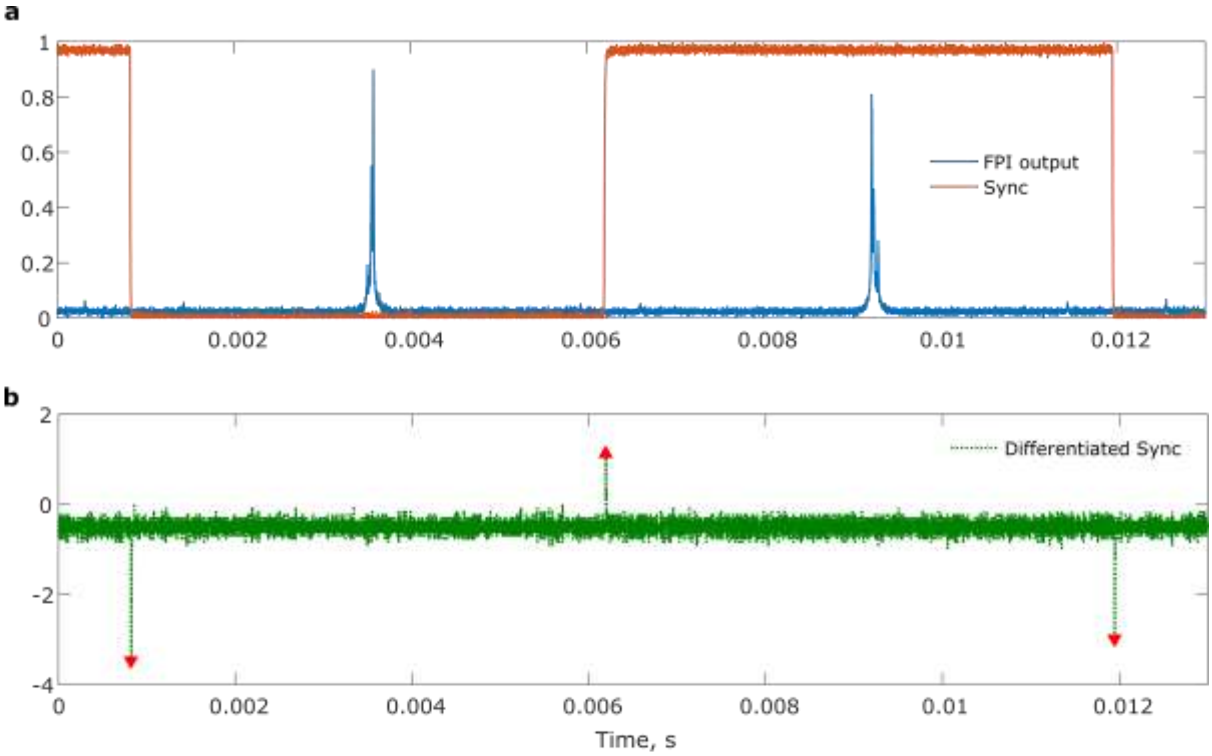

Supplementary Figure 2: Segmenting the spectrum-to-time mapped signal from the FPI. a. The sync signal (orange) obtained directly from the sweep generator is recorded with the FPI signal (blue). **b.** A simple first order difference of the sync signal results in a series of positive and negative peaks, giving the temporal instants the up and down-sweeps initiate.

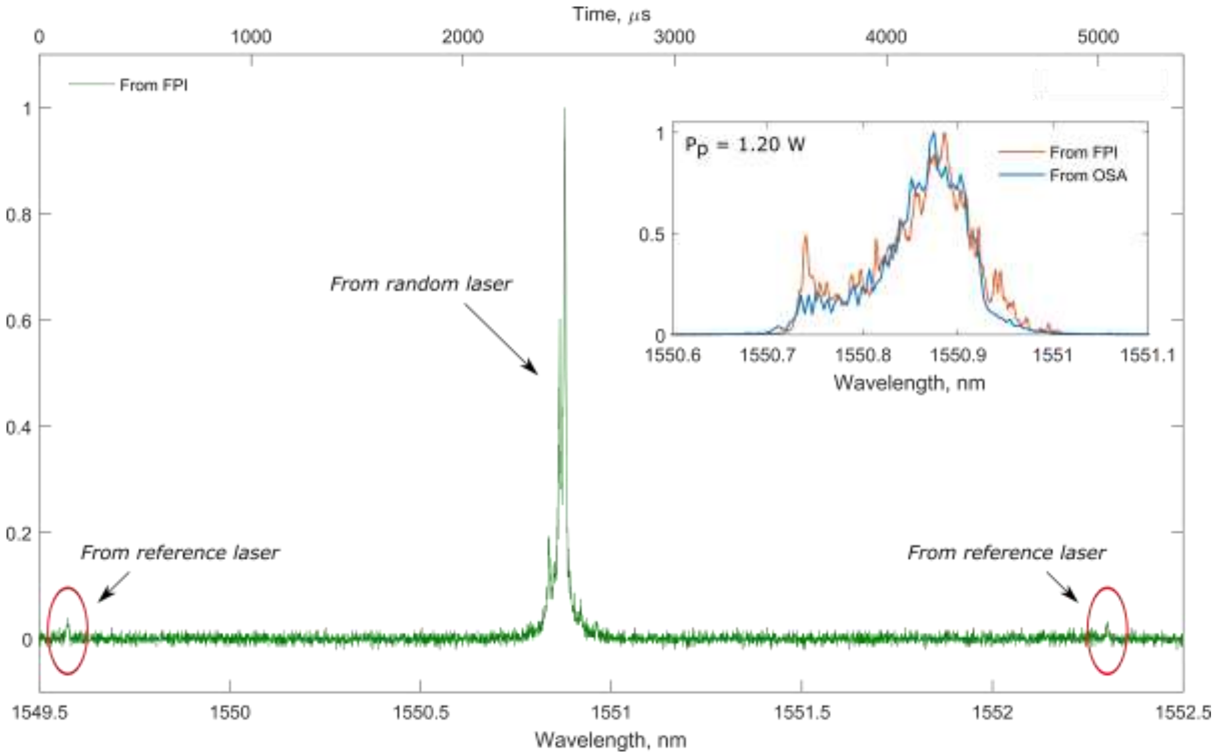

Supplementary Figure 3: Absolute Spectral calibration. Singe-shot FPI output (green curve) comprising of both random laser and reference laser output. Two instances of the reference laser are obtained, as the FPI sweep range is deliberately extended beyond the free spectral range (FSR). **Inset.** Comparison of measurements made with the FPI and a conventional optical spectrum analyzer.

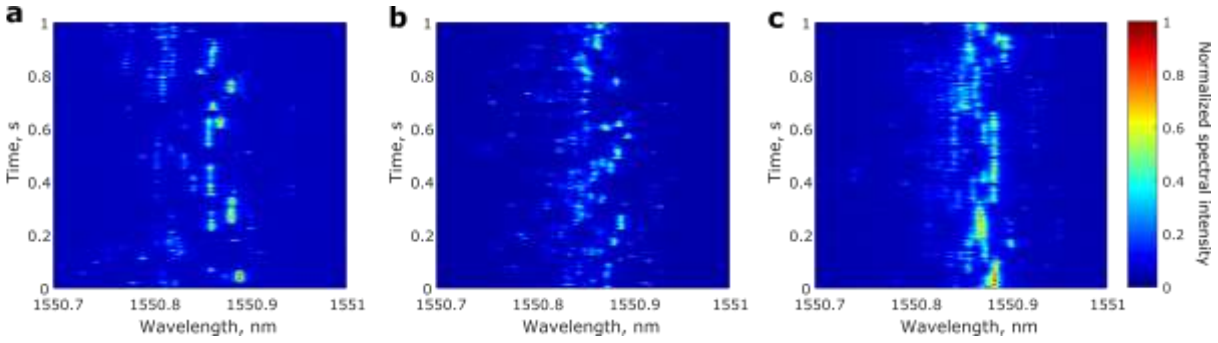

Supplementary Figure 4: Spectro-temporal evolution in the random fibre laser at different pump powers. **a.** 1.15 W, **b.** 1.18 W, **c.** 1.20 W.

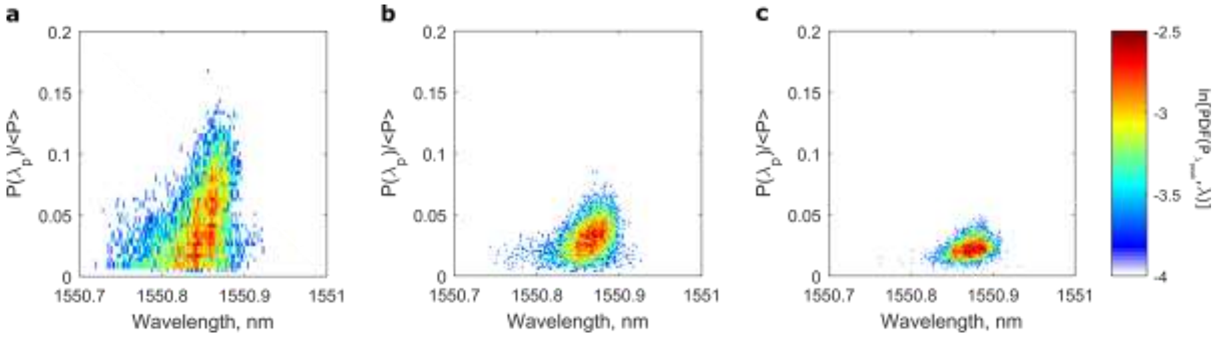

Supplementary Figure 5: Two-dimensional peak probability distribution functions in the random fibre laser at different pump powers. **a.** 1.15 W, **b.** 1.18 W, **c.** 1.20 W.

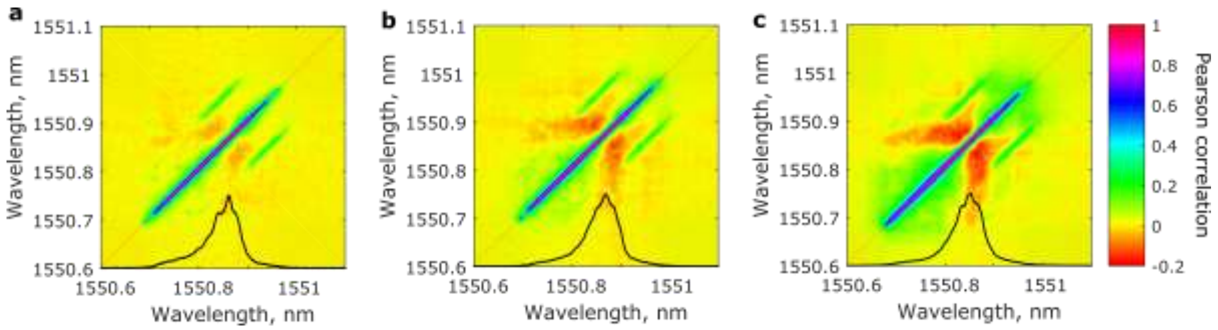

Supplementary Figure 6: Pearson correlation coefficient of the random fibre laser spectra at different pump powers. The black curve is the time-averaged FPI spectrum for the corresponding power. **a.** 1.15 W, **b.** 1.18 W, **c.** 1.20 W.

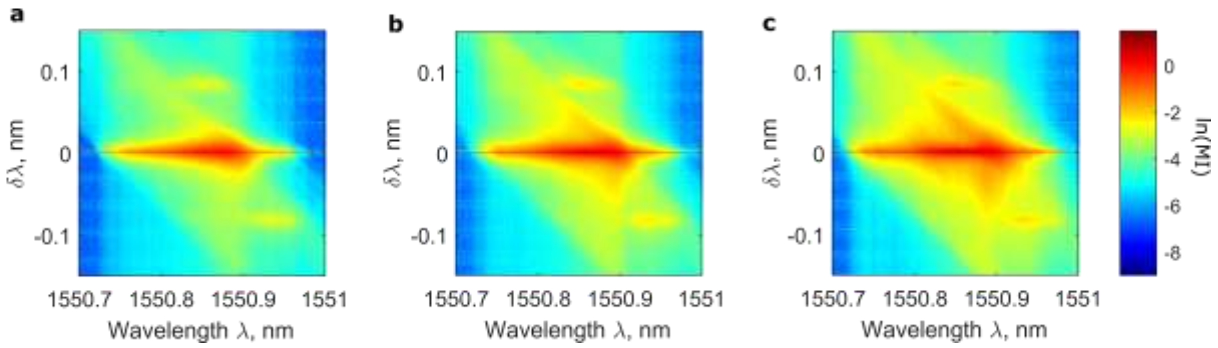

Supplementary Figure 7: Mutual information contour plots for the random fibre laser at different pump powers (logarithmic scale). **a.** 1.15 W, **b.** 1.18 W, **c.** 1.20 W.

Supplementary Note 1 – Spectral measurements using the scanning Fabry Perot Interferometer

In this note, we present step-by-step the procedure for calibrating the spectra measured using a scanning Fabry Perot Interferometer (FPI), and arriving at the real-time spectro-temporal dynamics as presented in Fig. 2 of the main article.

Fig S1 shows the experimental configuration used in the presented work. In principle, the random laser can be replaced with any light source. The output of the FPI is monitored using a photodetector-digital storage oscilloscope combination. Note that the FPI is preceded by a unidirectional optical isolator to mitigate feedback of FPI reflected components into the laser.

As the FPI samples the spectra during both its up and down sweeps (Fig S2a, blue curve), both sets of spectra can be used for the reconstruction of the spectro-temporal dynamics. This requires appropriate segmentation and registration of the output as continuously measured by the DSO. To achieve this, a synchronization signal can be derived from the FPI driver, which is then monitored simultaneously along with the FPI output (Fig S2a, orange curve). The exact starting time instants of the up and down sweeps can then be obtained from the sync-signal. In this work, these were obtained by differentiating the square-wave sync-signal derived from the FPI driver (Fig S2b). This information can then be used to segment the continuously recorded DSO signal. Note that the to-and-fro scanning action of the FPI results in the mirroring of the spectral features between consecutive sweeps. This spectral mirroring effect of the FPI can be corrected by temporally reversing every second spectral measurement, upon which successive spectral measurements can be stacked atop one another to arrive at the spectro-temporal representations as presented in Fig. 2 of the main article.

A reference is required to convert the time domain measurements to appropriate wavelength units. Towards this end, a narrow linewidth reference laser is also fed into the FPI along with random fibre laser. Fig S3 shows a single sweep instance of the measured FPI spectra (green curve), which reveals the reference laser output (encircled red). Here, the scan range of the FPI is deliberately extended beyond its free spectral range, enabling another transmission of the reference laser. The relation between wavelength and time units can then be expressed as [1]

$$\Delta\lambda = \frac{\lambda^2}{c}\left(\frac{\Delta f_{FSR}}{\Delta t_{FSR}}\right)\delta t \tag{1}$$

where λ is the wavelength of the reference laser, Δf_{FSR} is the free spectral range of the FPI, Δt_{FSR} is the temporal separation between the reference laser peaks, and δt is the sampling rate of the oscilloscope. The wavelength λ can then be further used express the relative wavelength units $\Delta\lambda$ in absolute terms. The inset of Fig. S3 shows appreciable agreement between the FPI spectra and that obtained using a conventional optical spectrum analyser (OSA). As the random fibre laser output was highly transient, the 'maximum-hold' measurement mode of the OSA was used, which retained only the largest peak excursion for each wavelength as obtained over a substantially long observation period (~mins). A similar maximum-hold signal was emulated from the FPI-measured spectro-temporal dynamics as depicted in Fig 2 of the main text, by retaining the highest peak excursion for each wavelength. The use of a reference laser increases measurement confidence, especially when characterizing a source exhibiting highly transient behaviour, like the random fibre laser here. Alternatively, if the spectral behaviour is more predictable, one may utilize averaged spectral measurements from both the FPI and the OSA and retrieve the spectral calibration factor using simple least-square fitting routines.

Supplementary References

[1] Hui, R., and O'Sullivan, M., *Fiber optic measurement techniques*. Academic Press, 2009.
